# Current News and Opinion

**Published:** 1885-07

**Authors:** 


### Current News and Opinion.

### WHAT IS DENTAL SURGERY?

We note that in nearly all of the States and in the District of Columbia laws are being enacted limiting the practice of "Dental Surgery" to a certain privileged class, which is designated as above. Now, if it is intended that physicians shall not treat any disease of the mouth unless they take out a degree from some dental "college," or from some medical school where there is

"annually delivered a full course of lectures and instructions in dentistry or dental surgery," as the Kansas dental law has it, it is about time that medical men were looking to their interests. The above law, however, generously permits "physicians, surgeons, or others," to extract teeth! If the dental "profession" has any claim to consider itself a specialty in medicine, this course is calculated to exclude it utterly. What would be thought if ophthalmologists, otologists or gynæcologists were to secure legislative enactment to prevent their medical colleagues from practicing in their field, unless they adopted the "profession" of ophthalmology, otology, or gynæcology, by taking out a "degree" in these respective branches? Such laws must be unconstitutional, or at least in conflict with the charters of medical schools, which are supposed to send out graduates empowered to treat any of the ills to which flesh is heir.—*Medical Record.*

Dreadful! Only think of it!! But really, *Mr. Medical Record,* is there anything so very strange in requiring practitioners of dentistry to show their qualifications for the practice of their specialty?

What proportion of physicians in the general practice of medicine are capable of performing such operations as dentists are called upon to perform? How many lectures are usually given to students of medical colleges in the course of a season, that relate to this specialty, even in the slightest degree? What opportunities have they to acquire a knowledge of these duties? The leading physicians in all our great cities admit that their knowledge of dentistry is exceedingly limited, and would as soon think of running a steam engine as to dabble at dentistry. And to a certain extent the same may be said of ophthalmology and general surgery. What proportion of the rank and file of "doctors of medicine" are thoroughly qualified to make nice operations on the eye, or to perform intricate surgical operations that require an advanced knowledge of anatomy, and a keen delicacy of touch that a skilled specialist in full practice has acquired? Perhaps the editor of the *Medical Record* is inflated with the notion that the degree of "M. D.," which he possesses, is the crowning degree of all knowledge, and indicates that its possessor is a sort of jack-of-all-trades. Individuals who *profess* to know too much, are not apt to show proficiency in anything. Many an "M. D." who has tried his hand at dentistry without proper training, has demonstrated the fact that the initials affixed to his name really meant "Miserable Dentist." Enough incompetent practitioners of dentistry have tortured humanity with their miserable operations. Why, then, object to the rooting out of charlatanism?

C. E. F.

### AMERICAN DENTAL ASSOCIATION.

The twenty-fifth annual meeting of the American Dental Association will be held at Minneapolis, commencing August 4, 1885. The present prospects are that the meeting will be an unusually large one. Railroad transportation has been secured at an unprecedentedly low rate. Tickets for the round trip from New York to Minneapolis and return, will be furnished for $24.00. Round trip from New York to Chicago and return, for $18.00. Round trip from Chicago to Minneapolis and return, $6.00.

At present it will be necessary for those wishing these tickets to secure them in Chicago. Later we may be able to make arrangements by which they can be secured at different points east. By sending a check for tickets to the chairman of the committee of arrangements, the tickets will be promptly forwarded. Negotiations are pending for rates from other points, and more definite information will be given in a circular sent to every member. The hotel rates are as follows : West Hotel, $4.00 per day. Nicollet House, $3.00. National Hotel, $2.00 per day. It is expected that a reduction from these prices will be made.

It is hoped that members having any new facts or ideas in regard to theory or practice, will come prepared to present them in connection with the section work. Any one having anything new in the way of appliances, will be given an opportunity to demonstrate its use during the half day that will be devoted to clinics.

### ATTRACTIONS AND EXCURSIONS.

Come equipped with guns and fishing tackle. While the interest and benefit of the meeting, the attractions of the trip and the beautiful city where we meet are too well known to need special mention, it may not occur to all that they will find themselves in one of the finest of hunting and fishing countries. Minnesota is especially famous for its prairie chicken and grouse shooting, and its fine fishing grounds. It is estimated that there are no less than 10,000 lakes dotting the State.

If any one wishes a still greater variety of scenery, to see a new, wild and picturesque country, to draw out the big brook trout, the black bass, the gamy pickerel, and the mighty muskallonge from the cold waters of the Lake Superior region, in fact, to enjoy the finest fresh water fishing in the world, a round trip ticket from

Chicago to Ashland and return will be furnished him for $10.00. A still greater attraction (if another were needed) is offered in the shape of a ten days' excursion to the far-famed Yellowstone National Park, immediately upon the close of the meeting, provided a sufficient number send in their names to warrant the securing of special cars and special rates. The committee believe that when so far on the way as Minneapolis, many will wish to avail themselves of this opportunity of seeing the grandest scenery in the world. The entire expense for the round trip from Minneapolis, including rail transportion, Pullman sleeping car fares, meals on Northern Pacific dining cars, hotel accommodations, five days in the Park, and stage transportation, will be $120.00. A circular describing the magnificent scenery in full, will be sent to every member of the American Dental Association at an early day. Others than members, who may contemplate going, will receive the same upon making application for it.

Come one, come all, and bring your wives along. It will be a trip that ladies will especially enjoy. Those wishing to go to Yellowstone Park will please send in their names at an early day, that all arrangements may be speedily and satisfactorily completed.

For further information address

J. N. CROUSE,

*Ch'n. of Com. of Arr.*

2101 Mich. Ave., Chicago, Ill.

---

PENNSYLVANIA STATE DENTAL SOCIETY.

The seventeenth annual meeting of the Pennsylvania State Dental Society will convene at Cresson Springs, Pa., Tuesday, July 28, 1885, at 10 A. M., and continue in session three days. Rates at the Mountain House have been reduced from four to three dollars per day to delegates and their families, dating from Saturday, July 25th, and continuing as long as desired. Orders for *special* excursion tickets will be issued over all lines of the Pennsylvania and A. V. R. R.'s. Usual excursion rates on other roads. Orders or general information can be obtained by addressing

W. H. FUNDENBERG,

958 Penn Ave., Pittsburg, Pa. *Cor. Sec.*

### NEW JERSEY STATE DENTAL SOCIETY.

The fifteenth annual session of the New Jersey State Dental Society will be held at the Coleman House, Asbury Park, commencing July 15th, and continuing in session three days. Every effort has been made to have this particular session the most memorable of any heretofore held. Interesting papers have been promised from the most eminent men in the profession, and the society membership have also contributed liberally. The clinics to be held will be made an important feature, and considerable time and attention has been given towards an exhibition of new and important surgical and mechanical appliances for use in dentistry. It is in contemplation to give a grand reception to the visiting members of the profession on one evening, to be arranged for. The place is easy of access from everywhere. The cuisine, the best; $2.50 per day, the rate. The location superb, within fifty feet of the surf. The hall for sessions, attached to the hotel, commodious and cool. All the profession will be made welcome. CHAS. A. MEEKER, D. D. S.,

*Secretary.*

### TO SUBSCRIBERS.

Complaints have occasionally been made that copies of the Independent Practitioner have miscarried, through defects in the address, or for some other reason. In copying so large a list each month, it was impossible always to avoid errors. To guard against them in the future we have had the subscription list printed, and the whole edition will in the future be directed from these mailing slips. The Buffalo office should have early notice of any failure in the receipt of each number. The Journal is regularly mailed on the last day of each month.

### SIXTH DISTRICT DENTAL SOCIETY.

The following is the list of officers elected at the sixteenth annual meeting of the Sixth District Dental Society :

*President*—C. E. Dunton, Cazenovia.

*Vice-President*—S. W. Ademy, Union.

*Secretary* —E. D. Downs, Owego.

*Treasurer*—Frank B. Darby, Elmira.

*Censor*—E. D. Downs, Owego.

*Delegates to State Society for four years*—W. C. Stewart, Elmira, A. M. Holmes, Morrissville.

### THE INTERNATIONAL MEDICAL CONGRESS.

At the late meeting of the American Medical Association, the authority granted to the committee appointed to invite the Congress to meet in Washington in 1887, and to make arrangements for the meeting, was virtually revoked, and a revisory committee was appointed. The original committee had been authorized by the Congress in session at Copenhagen, in 1884, to proceed with the arrangements, and when the invitation was accepted it became the committee—not of the American Medical Association, but of the International Congress,—and hence the American Association was wrong when it assumed to overrule and revise its action. If this were not so, then the Congress has no permanent existence and no continuing committees. But the "outs" were determined to become the "ins," and hence the mischievous action at New Orleans, which must prejudice the success of the whole meeting. The committee appointed at New Orleans is in session at the time of writing this paragraph, and a telegram informs us that it has dropped the section of Oral and Dental Surgery entirely. Probably it is as well, for dentists will be spared participation in a professional squabble that could not have proved entertaining or profitable.

---

### AMALGAM SOLVENTS.

In Dr. Francis' excellent article in the May number of the INDEPENDENT PRACTITIONER, occurs this assertion: "No solvent of amalgam pervades the precincts of the oral cavity." Properly inserted and well-polished amalgam fillings are to be seen with slight changes of condition. But the vast majority of amalgam fillings exhibit decided alteration in color, evidencing that something in the fluids of the mouth acts upon them. Now and then amalgam fillings are noticed, so acted upon as to have lost considerable material, the surfaces of the remaining portions appearing as would rust-eaten steel. Cases are presented in which this disintegration and roughness of amalgam fillings have caused discomfort to the patient. By the testimony of these scarred witnesses it would appear that a solvent of at least some grades of amalgam does pervade the oral cavity. B. H. TEAGUE.

---

### TO ADVERTISERS.

This journal will be found on file at Geo. P. Rowell & Co.'s Advertising Bureau, No. 10 Spruce Street, New York, and advertising contracts may there be made for it.

(63.) I wish some of the traveled readers of the I. P. would inform me whether, during a two months' vacation, it would be practicable for me to visit Europe with any degree of satisfaction, and what a trip would cost. Of course it will be too late for this year, but some of us, if we ever do go, must commence a long time in advance to make preparations. JUNIOR.

(64.) Can any reader of the "I. P." give any reason for the increase of Pyorrhœa Alveolaris, and will some one kindly state a course of treatment that has given satisfactory result? C. E. H. P.

(65.) We hear that much trouble is sometimes occasioned by exostosed or hypertrophied roots. I would like much to have some one well posted in histology inform me, through the INDEPENDENT PRACTITIONER, what produces exostosis, or how the ossific accretions are deposited? W.

(66.) I have heard or read of Tincture of Calendula as a therapeutic agent for dentists' use. Will some kindly disposed reader of your valuable journal please state its use, and how it can be obtained? "KANSAS."

(67.) Will some one please give in the next I. P. the process of uniting vulcanite and emery so that it may be vulcanized into one solid mass? A. M. T.

### Answers.

DENTOS. (61.) Bleaching teeth. The root canal should be well cleansed, carefully treated, and partly filled, that the apical foramen may be securely sealed. Cut away all the discolored dentine possible to spare; syringe with tepid water; dry out the cavity, then take a pellet of cotton moistened with Labarraque's Solution (Chlorinated Soda), gather upon it a quantity of finely powdered alum and pack it in the cavity. This rapidly generates chlorine gas, the agent needed for bleaching. This may be repeated several times, after which the cavity may again be washed and the walls lined with a thin coating of oxy-chloride of zinc. A case of two central incisors, recently treated in this manner, proved a decided success. C. E. F.

OLD DENTIST. (58.) In the *Dental Cosmos* for Sept., 1882, may be found a query of my own as follows:

"Some time since I made full upper and lower dentures for a gentleman aged 57. In wearing them he complains of the increased flow of saliva,—so much so that it will flow out of his mouth when speaking. Will some one inform me of the cause and give me a remedy for it? The upper plate is bow-spring and the lower weighted rubber. Would a celluloid plate be better?—BAYONNE."

In the October number of the same journal the following appears, and it may also benefit "Old Dentist," as it certainly did me.

"If 'Bayonne' will examine the case in question, he will propably find one or both his plates impinging on the salivary glands (presumably the lower on the sublingual), and by its constant irritation causing the flow. A plate should always be clear of the glands, and, with some exceptions, of the muscles also. If the muscles are allowed to bear on the plate, they tend to throw it off the jaw and make it unsteady." BAYONNE.

B. C. M. (53.) The treatment of the abscessed teeth of children is a difficult matter. Of course, nothing permanent can be done, and if they are in a very offensive, painful condition, it is practicable only to extract them. But if there is only a sub-acute inflammation, and if there is no very active discharge, it may be possible to cleanse the cavity of decay, as well as circumstances will permit, and to introduce some antiseptic, and when they are in a tolerable condition to fill with gutta-percha. I like Listerine very much for the treatment, as it is not unpleasant to the taste. When the tooth becomes comfortable the pulp chamber may be filled with cotton moistened with carbolic acid diluted with glycerine, and gutta-percha placed over that. The root canals may be filled as far as they can be easily reached, but the dentist must look to the extraction of the tooth when the permanent successor is ready to make its appearance, as the roots will not become absorbed as in the case of a living tooth. C. W. B.

